# The use of sivelestat sodium among patients with acute pancreatitis and early organ failure: a single-center retrospective cohort study

**DOI:** 10.3389/fmed.2026.1840457

**Published:** 2026-07-23

**Authors:** Zongwen Zhang, Xinsen Zou, Yiting Guan, Siyu Zhang, Mingfeng Huang, Hong Lin, Jing Zhou, Lin Gao, Wenjian Mao, Bin Yao, Xiangcheng Zhang, Lu Ke, Zhihui Tong, Yuxiu Liu, Weiqin Li

**Affiliations:** 1 Department of Critical Care Medicine, Center of Severe Acute Pancreatitis (CSAP), Jinling Hospital, Affiliated Hospital of Medical School, Nanjing University, Nanjing, Jiangsu, China; 2 Department of Intensive Care Unit, Bishan Hospital of Chongqing Medical University, Chongqing, China; 3 Department of Critical Care Medicine, School of Medicine, Center of Severe Acute Pancreatitis (CSAP), Southeast University, Nanjing, China; 4 Department of Critical Care Medicine, Center of Severe Acute Pancreatitis (CSAP), Jinling Hospital, Nanjing Medical University, Nanjing, Jiangsu, China; 5 National Institute of Healthcare Data Science, Nanjing University, Nanjing, Jiangsu, China; 6 Department of Intensive Care Unit, The Affiliated Huai’an No.1 People’s Hospital of Nanjing Medical University, Huai’an, Jiangsu, China; 7 Department of Biostatistics, School of Public Health, Nanjing Medical University, Nanjing, Jiangsu, China

**Keywords:** acute pancreatitis, early organ failure, inhibitor, neutrophil elastase, sivelestat sodium

## Abstract

**Background:**

This study aimed to investigate whether neutrophil elastase inhibitor (sivelestat sodium) was associated with decreased duration of organ failure in patients with acute pancreatitis (AP) and early organ failure.

**Methods:**

Between January 2022 and December 2024, patients who were diagnosed with AP and early organ failure were included. The primary outcome was organ failure-free days (OFFD) to Day 14 after admission. Propensity score matching (PSM) analysis was performed to control confounders. Multivariate zero-inflated negative binomial regression model was used to evaluate the association between sivelestat sodium and OFFD. An exploratory mediation analysis was carried out to evaluate whether day 3 C-reactive protein partly accounted for the association.

**Results:**

340 patients were enrolled in this study. After PSM, patients receiving sivelestat sodium had significantly more OFFD than those who did not (median [IQR], 9 [5 to 13] vs. 6 [1 to 10], *p* = 0.040). Using sivelestat sodium was associated with increased OFFD (Incidence rate ratio = 1.21, *p* = 0.001) after adjusting for covariates. The exploratory mediation analysis suggested that this association was primarily direct (1.28 days, *p* = 0.036), with a marginally significant component mediated through day 3 C-reactive protein reduction (0.13 days, *p* = 0.068).

**Conclusion:**

Sivelestat sodium use was associated with more organ failure-free days in patients with AP and early organ failure, particularly in those with respiratory failure. These findings require confirmation in future prospective randomized trials.

## Introduction

Acute pancreatitis (AP) is one of the most common gastrointestinal diseases that results in hospital admission worldwide. Approximately 8%–20% of AP cases develop early organ failure, with higher mortality and extended hospital length of stay (1–4). Although many efforts have been made to prevent or improve early organ failure, using supportive therapies like fluid resuscitation and early enteral nutrition as recommended by the current guidelines (4, 5), targeted therapeutics that address the underlying mechanisms of progressive organ failure are still limited.

Several studies have confirmed that activation and migration of neutrophils play an important role in the progression of remote organ failure during the first week of AP onset (6, 7). Upon activation, neutrophils are recruited to remote organs via chemokine signaling and release neutrophil elastase (NE), a potent serine protease with direct cytotoxic effects. NE can directly degrade extracellular matrix components, resulting in tissue damage and contributing to early organ failure in AP (8, 9). Moreover, NE can activate key cytokines [e.g., interleukin (IL)-6] and stimulate neutrophil extracellular traps (NETs) formation, which can also aggravate tissue damage. Previous studies have confirmed that elevated plasma NE concentration and activity are associated with severity and mortality in AP (10, 11), supporting that NE could be a potential therapeutic target. Accordingly, inhibition of NE has been evaluated to reduce AP-induced organ failure in animal experiments (12–14).

The NE inhibitor, sivelestat sodium, has been reported to be safe and effective in treating acute lung injury and acute respiratory distress syndrome (ARDS) in a number of studies (15–18). Nevertheless, current evidence predominantly comes from preclinical and cohort studies of other inflammatory conditions, whereas clinical studies specifically examining NE inhibition in AP patients with early organ failure are scarce. This study aimed to investigate whether the neutrophil elastase inhibitor (sivelestat sodium) was associated with decreased duration of organ failure in AP patients with early organ failure.

## Materials and methods

### Study design

This single-center retrospective cohort study utilized prospectively collected data from the Center of Severe Acute Pancreatitis (CSAP) in the Department of Critical Care Medicine, Jinling Hospital, Nanjing, China. All clinical and laboratory data were extracted from the electronic database with the approval of the ethics committee of Jinling Hospital, Nanjing University (2019NZKY-003-01). Written informed consent was obtained from all participants or their next of kin for academic use. The study was carried out in accordance with current revision of the 1964 Helsinki Declaration and with the laws and regulations of the nation.

### Patients and definition

Between January 2022 and December 2024, patients who were diagnosed with AP and early organ failure and admitted within 72 h of abdominal pain onset were enrolled in this study. Exclusion criteria included: (1) age < 18 years, (2) pregnancy/lactation, (3) pre-existing chronic organ failure or active malignancy.

The primary outcome was organ failure-free days (OFFD) within the first 14 days after admission, defined as the number of consecutive days alive and free from organ failure after the final resolution of organ failure, based on daily Sequential Organ Failure Assessment (SOFA) score, which has been described in previous study (19–21). An individual SOFA score of 2 or more in any of cardiovascular, respiratory, or renal organ system was defined as organ failure. For each day, patients who were alive and did not meet the criteria for organ failure received one OFFD. For patients who were alive, free from organ failure, and discharged from the hospital before Day 14, OFFD was counted from the day of discharge. Patients who died before Day 14, or whose next of kin withdrew treatment due to clinical deterioration before organ failure had resolved, were assigned an OFFD of 0. Cardiovascular OFFD, respiratory OFFD and renal OFFD were also counted using the same approach.

Secondary outcomes included the presence of organ failure at day 7 after admission, sivelestat sodium using time, sivelestat sodium using dose, length of hospital stay, length of intensive care unit (ICU) stay, the presence of pancreatic necrosis, and 28-day mortality. Sivelestat sodium using time was defined as the number of days that sivelestat sodium was used during the first hospitalization. Sivelestat sodium using dose was defined as the total dosage of sivelestat sodium used during the first hospitalization. Hepatic dysfunction was defined as ALT or AST elevation above the upper limit of normal during hospitalization. Mild ALT/AST elevation was defined as ALT or AST elevation above the upper limit of normal but not exceeding three times the upper limit of normal. Thrombocytopenia was defined as a platelet count below 100 × 10^9^/L.

### Treatment strategy

The majority of patients admitted between January 2022 and July 2023 did not receive sivelestat sodium. From August 2023 to December 2024, the potential role of sivelestat sodium for AP-related lung injury were increasingly recognized. The entire cohort was divided into two groups: sivelestat sodium group and non-sivelestat sodium group. Patients in the sivelestat sodium group received sivelestat sodium 4.8 mg/kg/day intravenously according to the drug label. Sivelestat sodium was used for patients after documentation of at least one organ failure (respiratory, renal, or cardiovascular) as defined by the SOFA score, but within 24 h of organ failure diagnosis (22). All the patients were managed by the same team throughout the study period according to the international guidelines (4, 5, 23).

### Data collection and missing data handling

Demographic characteristics and baseline clinical features, including age, gender, etiology, Revised Atlanta classification (RAC), body mass index (BMI), intervals from AP onset to admission, presence of organ failure, computed tomography severity index (CTSI), SOFA score, acute physiology and chronic health evaluation (APACHE) II score, were prospectively collected. The treatment patients received was collected, including fluid resuscitation, nutritional support, antibiotics use, mechanical ventilation (MV), continuous renal replacement therapy (CRRT) and vasopressor use. MV, CRRT, and vasopressor use included in the analysis were already present before sivelestat sodium use. In addition, laboratory indexes including white blood cell (WBC), C-reactive protein (CRP), procalcitonin (PCT), and IL-6 were also collected. Adverse events related to sivelestat sodium, including hepatic dysfunction, renal dysfunction, thrombocytopenia, bleeding, allergic reactions, secondary infections, and treatment discontinuation, were also recorded. The multiple imputation method was used for variables with missing values of less than 20%.

### Statistical analysis

The continuous variables were reported as median (interquartile range, IQR), whereas categorical variables were reported as frequencies and percentages. Continuous variables were compared using the Mann-Whitney U test, and categorical data were analyzed with chi-square test or Fisher’s exact test.

A 1:1 propensity score matching (PSM) analysis was performed to control potential confounders, using the nearest neighbor method with the caliper width set to 0.2 of the standard deviations of the logit of the propensity score. Standardized mean difference (SMD) was used to assess the balance of baseline covariates between the two matched groups. A SMD of more than 0.1 and a 2-sided *p*-value of less than 0.05 indicated a significant imbalance in the baseline covariate. Three rules were followed to choose the variables for PSM: (1) differences between groups with a *p*-value less than 0.1 or SMD more than 0.2; (2) potentially relevant variables according to previous studies and clinical considerations; and (3) missing data less than 10%. Because the sivelestat group included only 60 patients, we limited the number of covariates in the PSM model to avoid overfitting and unstable matching, according to the 10 events-per-variable heuristic. As a result, etiology, the presence of organ failure at admission, and baseline SOFA score were involved. Group differences in the PSM cohort were compared using the Wilcoxon signed-rank test and McNemar’s test for matched data.

In addition, given the substantial proportion of patients with OFFD of 0 (15%) and overdispersion of the data, a zero-inflated negative binomial regression model was used to evaluate the association between sivelestat sodium use and OFFD to 14 days after admission. The zero-inflated negative binomial regression model was selected based on Vuong’s test, which confirmed its superior fit over the standard negative binomial model (z = −4.879, *p* < 0.001; AIC: 1798.3 vs. 1971.3). Incidence rate ratio (IRR) followed by 95% confidence interval (CI) was calculated.

To evaluate the robustness of our findings, we performed two sensitivity analyses. For the first, unstabilized inverse probability of treatment weighting (IPTW) was applied using etiology, organ failure at admission, and baseline SOFA score. After IPTW, the association between sivelestat sodium use and OFFD was further analyzed using a weighted zero-inflated negative binomial regression model, additionally adjusting for age, BMI, APACHE II score, CTSI, WBC, CRP, PCT, and IL-6. For the second, given collinearity between pre-treatment organ support therapies and baseline organ failure, we excluded baseline organ failure variables and instead included three organ support therapies as covariates.

An exploratory mediation analysis was conducted to evaluate whether day-3 CRP accounted for the association between sivelestat sodium use and OFFD. The total effect and direct effect of the association between using sivelestat sodium and OFFD were calculated, as well as the indirect effect and mediation proportion of the inflammatory marker.

Statistical analyses were performed using R software (version 4.4.3, Alcatel-Lucent Bell Labs, New Jersey, United States). A two-sided *p*-value of less than 0.05 was considered statistically significant.

## Results

### Patient baseline characteristics

**FIGURE 1:**
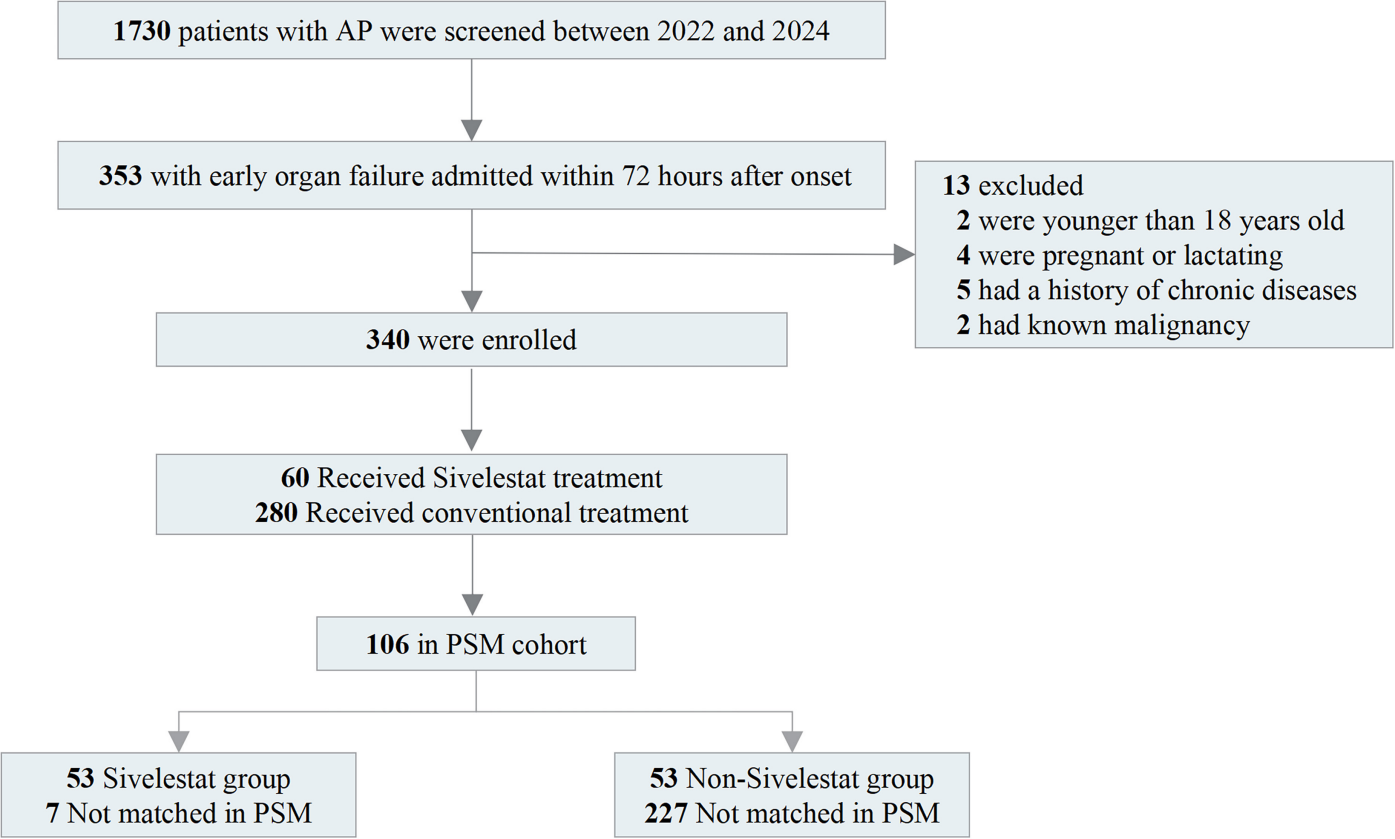
Study flowchart.

Between January 2022 and December 2024, 1,730 patients were diagnosed with AP (Figure 1). A total 340 patients were enrolled in the final analysis. Among them, 60 patients received sivelestat sodium (sivelestat group) and 280 patients did not (non-sivelestat group). Demographics and baseline clinical features of the two groups are shown in Table 1. Baseline cardiovascular failure (9 [15.00%] vs. 11 [3.90%]; *p* = 0.003) and respiratory failure (52 [86.70%] vs. 167 [59.64%]; *p* < 0.001) were more frequent in the sivelestat group. Baseline SOFA score in Sivelestat group was higher than that in non-sivelestat group. All 340 patients received adequate fluid resuscitation, early enteral nutritional support, and prophylactic antibiotics.

**TABLE 1:** Demographics and Baseline characteristics in the study cohort before and after propensity score matching.

Variables	Before PSM			After PSM		
	Sivelestat group (*N* = 60)	Non-sivelestat group (*N* = 280)	*P*-value	Sivelestat group (*N* = 53)	Non-sivelestat group (*N* = 53)	*P*-value
Age, year	40 (36–51)	40 (32–50)	0.366	40 (36–52)	42 (35–51)	0.830
Gender, *n* (%)	–	–	0.985	–	–	0.367
Male	43 (71.67)	201 (71.79)	–	38 (71.70)	42 (79.25)	–
Female	17 (28.33)	79 (28.21)	–	15 (28.30)	11 (20.75)	–
Etiology, *n* (%)	–	–	0.428	–	–	0.652
Biliary	14 (23.33)	65 (23.21)	–	12 (22.64)	14 (26.42)	–
Hyperlipidemia	46 (76.67)	202 (72.14)	–	41 (77.36)	39 (73.58)	–
Alcohol	0	12 (4.29)	–	–	–	–
Other	–	1 (0.36)	–	–	–	–
RAC, *n* (%)	–	–	0.716	–	–	0.174
MSAP	17 (28.33)	86 (25.29)	–	16 (30.18)	11 (20.76)	–
SAP	43 (71.67)	194 (74.71)	–	37 (69.81)	42 (79.24)	–
Body mass index	27.33 (25.24–29.39)	27.55 (24.69–30.04)	0.898	27.68 (25.39–29.41)	27.04 (23.80–29.39)	0.255
Intervals from AP onset to admission, d	2 (1–3)	2 (1–3)	0.879	2 (1–3)	2 (2–3)	0.671
CTSI	5 (4–6)	6 (4–7)	0.268	5 (4–6)	6 (3–8)	0.183
Organ failure on admission, n (%)						
Cardiovascular failure	9 (15.00)	11 (3.90)	**0.003**	5 (9.43)	4 (7.55)	1.000
Respiratory failure	52 (86.70)	167 (59.64)	<**0.001**	45 (84.91)	44 (83.02)	0.791
Renal failure	14 (23.30)	39 (13.93)	0.068	10 (18.87)	9 (16.98)	0.800
SOFA score	5 (3–7)	3 (2–4)	<**0.001**	5 (3–6)	4 (3–6)	0.957
APACHE II score	7 (4–11)	8 (5–10)	0.136	5 (4–10)	7 (3–9)	0.687
WBC	11.1 (7.6–13.7)	11.2 (8.4–14.2)	0.442	11.3 (7.7–14.5)	10.8 (8.3–13.2)	0.571
CRP	203.8 (139.6–252.6)	207.5 (153.8–258.0)	0.512	203.4 (133.8–262.2)	190.5 (123.2–235.2)	0.376
PCT	1.8 (0.5–4.3)	1.1 (0.4–3.7)	0.252	1.6 (0.4–3.3)	1.9 (0.5–4.8)	0.066
IL-6	158.2 (84.9, 281.7)	153.40 (84.0, 287.5)	0.923	154.8 (73.5–290.8)	141.5 (70.2–240.0)	0.955
MV, *n* (%)	12 (20.00)	31 (11.10)	0.059	8 (15.10)	8 (15.10)	1.000
CRRT, *n* (%)	10 (16.67)	30 (10.71)	0.194	6 (11.32)	6 (11.32)	1.000
Vasopressor use, *n* (%)	7 (11.67)	27 (9.64)	0.631	4 (7.55)	5 (9.43)	1.000

RAC, Revised Atlanta classification; MAP, moderate acute pancreatitis; SAP, severe acute pancreatitis; CTSI, computed tomography severity index; SOFA, Sequential Organ Failure Assessment; APACHE, acute physiology and chronic health evaluation; WBC, white blood cell; CRP, C-reactive protein; PCT, procalcitonin; IL, interleukin; MV, mechanical ventilation; CRRT, continuous renal replacement therapy. Bold values denote statistical significance (*p* < 0.05).

After PSM, 53 matched pairs were generated. The imbalance in the baseline characteristics was significantly reduced after PSM (Supplementary Table 1 and Supplementary Figure 1). The baseline characteristics of the PSM cohort are shown in Table 1. There was no significant difference between groups after PSM.

### Clinical outcomes in the matched cohort

**FIGURE 2:**
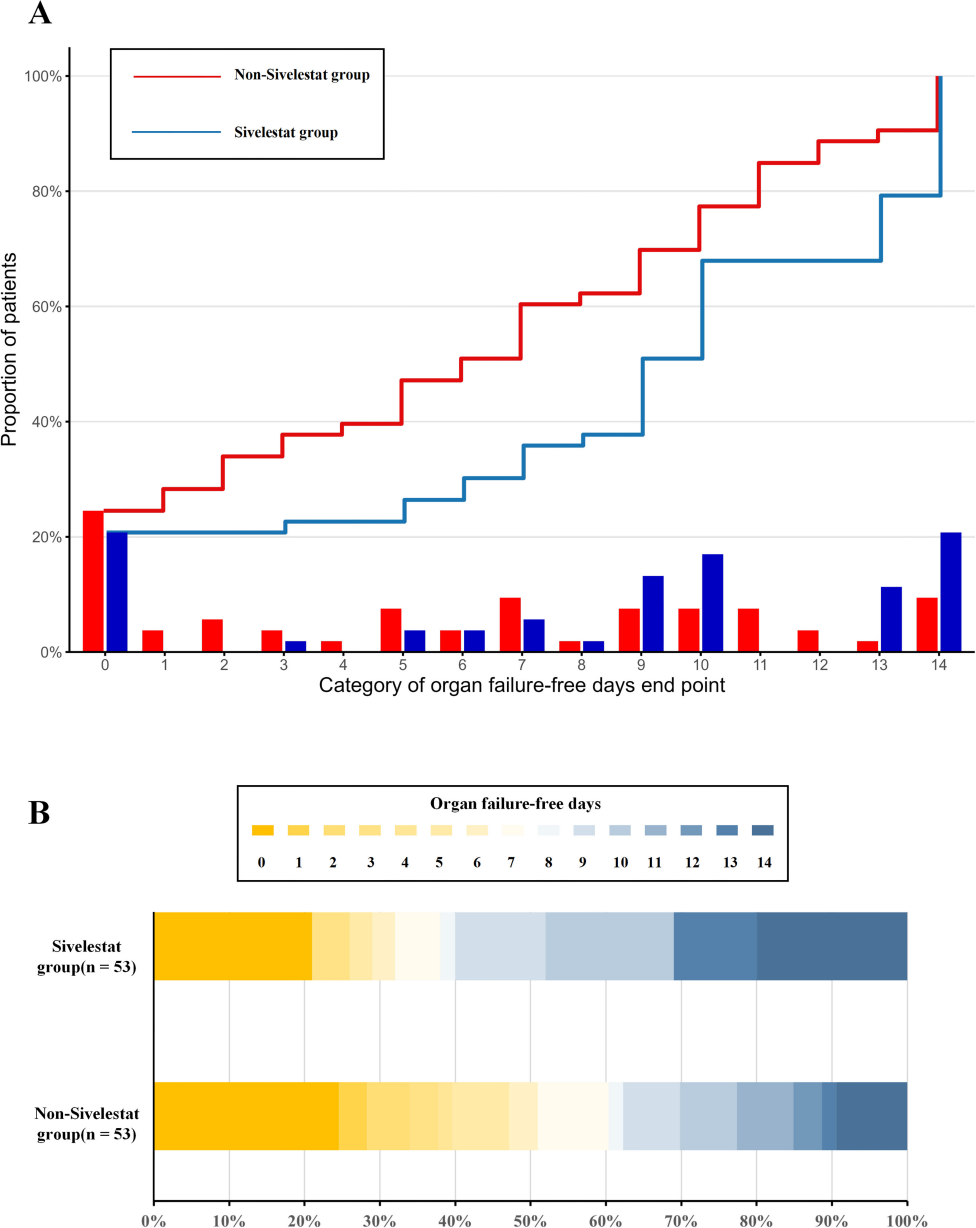
The distribution of organ failure-free days to Day 14 in the matched groups. **(A)** Provides the ordinal scale a score of 0–14 (the number of days alive and without organ failure) as the cumulative proportion (y-axis) for each group by day (x-axis). **(B)** Provides a visual comparison of outcomes using color-coded bars: blue shades indicate more organ failure-free days (better outcomes), and yellow shades indicate fewer days (worse outcomes). The darkest blue corresponds to 14 full days without organ failure, while the darkest yellow indicates zero days.

The clinical outcomes in the matched cohorts are shown in Table 2. For primary outcomes, patients in the sivelestat group had significantly more OFFD to Day 14 after admission than those in the non-sivelestat group (median [IQR], 9 [5 to 13] vs. 6 [1 to 10], *p* = 0.040). The distribution of OFFD of the matched cohorts is shown in Figure 2. Among the three organ systems, only respiratory OFFD was significantly different between groups (median [IQR], 9 [6 to 13] vs. 6 [1 to 10], *p* = 0.029). For secondary outcomes, the number of patients who had organ failure in Day 7 in the sivelestat group was significantly lower than that in the non-sivelestat group (35.85% vs. 60.38%, *p* = 0.011). The sivelestat group had a lower incidence of day 7 respiratory failure compared to the non-sivelestat group (33.96% vs. 58.49%, *p* = 0.015). In addition, there was no difference in other outcomes between the two matched cohorts.

**TABLE 2:** Clinical outcomes after propensity score matching.

Variables	Sivelestat group (*N* = 53)	Non-sivelestat group (*N* = 53)	*P*-value
Primary outcomes			
OFFD to Day 14, d	9 (5–13)	6 (1–10)	**0.040**
Cardiovascular OFFD	14 (14–14)	14 (14–14)	0.305
Respiratory OFFD	9 (6–13)	6 (1–10)	**0.029**
Renal OFFD	14 (14–14)	14 (14–14)	0.621
Secondary outcomes			
Organ failure at day 7, *n* (%)	19 (35.85)	32 (60.38)	**0.011**
Cardiovascular failure	6 (11.32)	2 (3.77)	0.219
Respiratory failure	18 (33.96)	31 (58.49)	**0.015**
Renal failure	9 (16.98)	10 (18.87)	1.000
Sivelestat using time, d	5 (2–7)	–	–
Sivelestat using dose, g	1.5 (0.8–2.1)	–	–
Length of stay, d	7 (5–15)	9 (7–16)	0.774
ICU length of stay, d	5 (2–9)	7 (4–14)	0.533
Pancreatic necrosis, *n* (%)	30 (56.60)	33 (62.26)	0.832
28-day mortality, *n* (%)	1 (1.89)	3 (5.66)	0.625

OFFD, organ failure-free days; ICU, intensive care unit. Bold values denote statistical significance (*p* < 0.05).

### Multivariable and mediation analyses

In the multivariable zero-inflated negative binomial model, using sivelestat sodium was associated with increased OFFD (IRR = 1.21, 95% CI 1.08–1.36, *p* = 0.001) after adjusting for covariates (Table 3). This association remained significant in the unstabilized IPTW-weighted zero-inflated negative binomial sensitivity analysis (IRR, 1.13; 95% CI, 1.06–1.20; *p* < 0.001; Supplementary Table 2). Likewise, after excluding baseline organ failure variables and adjusting for MV, CRRT, and vasopressor use, the association remained significant (IRR = 1.19, 95% CI: 1.06–1.33, *p* = 0.002; Supplementary Table 3).

**TABLE 3:** Risk factors associated with organ failure-free days to Day 14 identified by multivariate zero-inflated negative binomial model.

Variables	IRR (95% CI)	*P*-value
Group-sivelestat administration	1.21 (1.08–1.36)	**0.001**
Treatment era	0.95 (0.86–1.05)	0.299
Gender-male	0.90 (0.82–0.99)	**0.027**
Age	1.00 (0.99–1.00)	0.390
Body mass index	1.00 (0.99–1.01)	0.973
CTSI	0.99 (0.97–1.01)	0.285
Organ failure on admission		
Cardiovascular failure	1.50 (1.03–2.18)	**0.035**
Respiratory failure	1.02 (0.79–1.33)	0.864
Renal failure	0.86 (0.77–0.96)	**0.006**
SOFA score	0.93 (0.90–0.97)	**<0.001**
APACHE II score	0.99 (0.98–1.00)	0.239
CRP	0.99 (0.99–1.00)	**0.037**

CTSI, computed tomography severity index; SOFA, Sequential Organ Failure Assessment; APACHE, acute physiology and chronic health evaluation; CRP, C-reactive protein, IRR, incidence rate ratio. Bold values denote statistical significance (*p* < 0.05).

**FIGURE 3:**
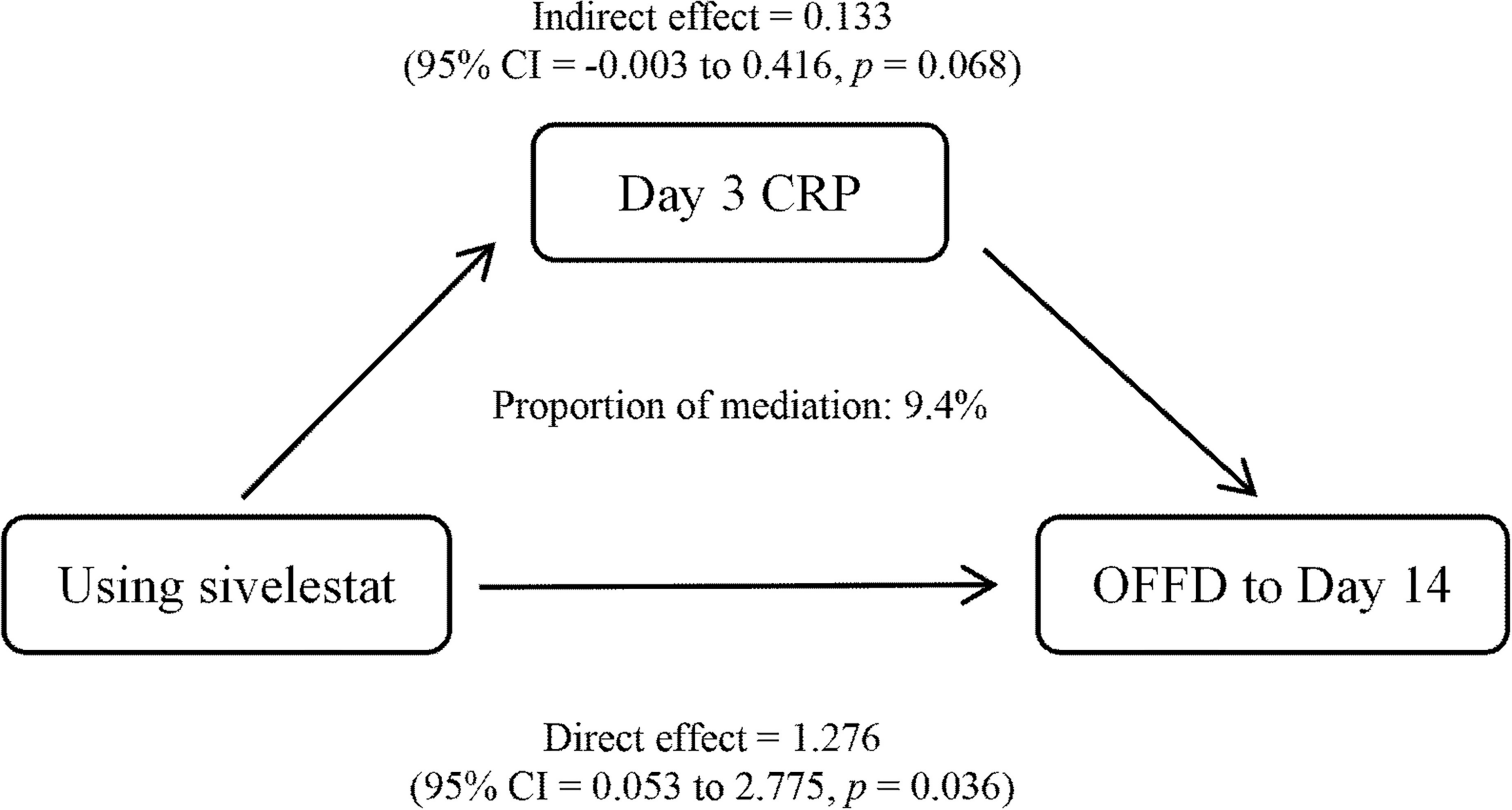
Mediation analysis between using sivelestat sodium and organ failure-free days to Day 14.

Given substantial missing data in other inflammatory markers (Supplementary Table 4), D3 CRP (8.5% missing) was chosen as the primary mediator for multiple imputation and subsequent mediation analysis. The exploratory mediation analysis revealed that using sivelestat sodium was associated with higher OFFD (total effect = 1.41 days, 95% CI, 0.15 to 2.92, *p* = 0.032) (Figure 3). This association was primarily direct (1.28 days, 95% CI, 0.05 to 2.78, *p* = 0.036), with a smaller, marginally significant component mediated through Day 3 CRP reduction (0.13 days, 95% CI −0.003 to 0.38, *p* = 0.068). CRP reduction accounted for approximately 9.4% of the total treatment effect (95% CI, 1.2% to 39.3%, *p* = 0.092).

### Safety data

Table 4 summarized adverse events considered by treating clinicians to be possibly related to sivelestat sodium use, whereas Supplementary Table 5 presented the overall occurrence of adverse events in both groups regardless of attribution. In sivelestat group, mild ALT or AST elevation was observed in nine patients (15.0%) and mild thrombocytopenia in five patients (8.3%). All cases resolved with appropriate treatment, and no serious adverse events, bleeding, allergic reactions, secondary infections, or treatment discontinuations were observed.

**TABLE 4:** Adverse events related to sivelestat sodium in 60 patients.

Variables	Sivelestat group (*N* = 60)
Hepatic dysfunction, *n* (%)	9 (15)
Renal dysfunction, *n* (%)	0
Thrombocytopenia, *n* (%)	5 (8.3)
Bleeding, *n* (%)	0
Allergic reactions, *n* (%)	0
Secondary infection, *n* (%)	0
Treatment discontinuation, *n* (%)	0

## Discussion

In this single-center retrospective cohort study, we found that the use of sivelestat sodium was associated with a significant increase in OFFD to Day 14 after admission among patients with AP and early organ failure after adjustment for covariates. Among the three major organ systems, sivelestat sodium use was associated with higher respiratory OFFD and a lower incidence of respiratory failure at day 7, possibly related to lower systemic inflammation.

These results are consistent with a prior retrospective study that also reported favorable associations of sivelestat sodium in severe AP patients with systemic inflammatory response syndrome (24), including shorter duration of mechanical ventilation, accelerated renal recovery, and reduced ICU length of stay. Our findings further support that association between sivelestat sodium use and higher OFFD in patients with AP and early organ failure, particularly in the respiratory system, reinforcing its potential pulmonary protective effect across a broader clinical population. The observed association of sivelestat sodium on OFFD, a composite endpoint increasingly recognized for its clinical relevance in critical illness trials as it captures both mortality and organ dysfunction burden, is particularly noteworthy. This OFFD definition emphasizes sustained organ function recovery rather than cumulative transient organ failure-free time. Because organ function status was assessed using the SOFA score before discharge, patients discharged early were not automatically considered organ failure-free without clinical evaluation. However, our study did not observe significant differences in ICU length of stay or renal outcomes. This discrepancy may be explained by differences in patient populations, including baseline disease severity or comorbidities.

Mechanistically, NE plays a dual role: while it contributes to host defense through bacterial killing and degradation of toxins via NETs, its excessive release promotes inflammation by upregulating cytokine expression and disrupting endothelial barrier integrity, thereby exacerbating tissue edema and inflammatory cascades (25–29). As a selective competitive inhibitor of NE, sivelestat sodium is clinically approved in China for acute lung injury and ARDS (15), offering a targeted pharmacologic option in patients complicated by respiratory failure. The observed association in AP may plausibly relate to interruption of neutrophil-driven injury, a key pathway in AP-related systemic inflammation and distant organ failure (10, 11).

A key and compelling aspect of our results is the specificity of the observed respiratory association. Using sivelestat sodium was associated not only with increased respiratory-specific OFFD but also with a significant reduction in the incidence of respiratory failure specifically at day 7 after admission, suggesting that NE inhibition may be relevant to respiratory failure in patients with AP. The lungs are highly vulnerable to neutrophil-mediated injury due to their extensive microvascular network and exposure to circulating inflammatory mediators (“first-pass” effect). NE directly damages pulmonary endothelial and epithelial cells, increases vascular permeability, impairs surfactant function, and promotes alveolar edema. These processes collectively represent key pathological features of ARDS, a common complication in the early phase of AP. Our findings were consistent with the known role of NE in ARDS pathogenesis and the rationale for targeting it.

The exploratory mediation analysis suggested that the association between sivelestat sodium use and OFFD was predominantly direct, with only a modest and marginally significant contribution from day 3 CRP reduction. Given that CRP is a non-specific inflammatory marker and does not directly represent neutrophil elastase inhibition, this analysis should be interpreted as exploratory and hypothesis-generating. These findings are consistent with the possibility that sivelestat sodium use may be related to organ protection, but confirmatory studies using more specific biomarkers of neutrophil activity are warranted.

This study also had some limitations. First, due to the retrospective design, the observed association cannot be interpreted as evidence of a direct drug effect, despite adjustment for the treatment era and other important covariates in the regression models. The association may partly reflect temporal changes in clinical practice, including variations in supportive care and evolving management strategies over time, rather than the effect of sivelestat sodium. Second, although the sample size was sufficient to find differences in OFFD, it may have been underpowered for mortality outcomes. Third, the study was conducted at a single tertiary center with a high proportion of hypertriglyceridemia-induced acute pancreatitis, and the majority of patients had early organ failure, particularly respiratory failure. Therefore, our findings may not be directly generalizable to patients with other etiologies, later-onset organ failure, or different clinical settings. Fourth, the mediation analysis was exploratory, and the marginal indirect association through day-3 CRP should not be interpreted as evidence of a confirmed biological mechanism. We did not collect biomarkers specifically related to neutrophil elastase activity or NETosis (e.g., citrullinated histone H3, or cell-free DNA), which would be more suitable than CRP for elucidating the mechanistic pathway of sivelestat sodium and identifying potential treatment responders.

## Conclusion

In conclusion, this study shows that the use of sivelestat sodium was associated with more organ failure-free days in patients with AP and early organ failure, particularly in those with respiratory failure. Future prospective, randomized controlled trials targeting AP patients with early organ failure are warranted to confirm these findings.

## Data Availability

The raw data supporting the conclusions of this article will be made available by the authors, without undue reservation.
