# Supplementary material for: The use of sivelestat sodium among patients with acute pancreatitis and early organ failure: a single-center retrospective cohort study
Source: Front Med (Lausanne). 2026 Jul 23;13:1840457. doi: 10.3389/fmed.2026.1840457 (PMC13441825; doi:10.3389/fmed.2026.1840457)
Supplement: Supplementary file 1 [file Table_1.docx]

**Supplementary Table 1. Standardized mean differences before and after propensity score matching.**

| **Variables** | SMD before PSM | SMD after PSM |
| --- | --- | --- |
| Age | 0.092 | 0.062 |
| Gender: Male | 0.003 | 0.175 |
| Etiology |  |  |
| Biliary | 0.003 | 0.088 |
| Hyperlipidemia | 0.104 | 0.088 |
| Alcohol | 0.296 | 0.000 |
| other | 0.085 | 0.000 |
| RAC: SAP | 0.052 | 0.217 |
| Body Mass Index | 0.050 | 0.233 |
| Intervals from AP onset to admission | 0.018 | 0.070 |
| CTSI | 0.142 | 0.246 |
| Organ Failure on admission |  |  |
| Cardiovascular failure | 0.378 | 0.068 |
| Respiratory failure | 0.610 | 0.051 |
| Renal failure | 0.242 | 0.049 |
| SOFA Score | 0.701 | 0.017 |
| APACHE II score | 0.128 | 0.079 |
| WBC | 0.081 | 0.109 |
| CRP | 0.102 | 0.116 |
| PCT | 0.151 | 0.251 |
| IL-6 | 0.047 | 0.145 |
| MV | 0.246 | 0.000 |
| CRRT | 0.173 | 0.000 |
| Vasopressor use | 0.066 | 0.068 |
| PSM, propensity score matching; RAC, revised Atlanta classification; SAP, severe acute pancreatitis; CTSI, computed tomography severity index; SOFA, Sequential Organ Failure Assessment; APACHE, Acute Physiology and Chronic Health Evaluation; WBC, white blood cell; CRP, C-reactive protein; PCT, procalcitonin; IL, interleukin; MV, mechanical ventilation; CRRT, continuous renal replacement therapy. | | |

**Supplementary Table 2. Sensitivity analysis for the organ failure-free days to Day 14 using unstabilized IPTW-weighted zero-inflated negative binomial regression.**

| **Variables** | **IRR (95% CI)** | ***p* value** |
| --- | --- | --- |
| Group-Sivelestat administration | 1.13 (1.06- 1.20) | **＜0.001** |

The weighted zero-inflated negative binomial regression model adjusted for etiology, baseline organ failure, and baseline SOFA score, age, BMI, APACHE II score, CTSI, WBC, CRP, PCT, and IL-6.

IPTW, inverse probability of treatment weighting; IRR, incidence rate ratio; CI, confidence interval; SOFA, Sequential Organ Failure Assessment; BMI, body mass index; APACHE, Acute Physiology and Chronic Health Evaluation; CTSI, computed tomography severity index; WBC, white blood cell; CRP, C-reactive protein; PCT, procalcitonin; IL, interleukin.

**Supplementary Table 3. Sensitivity analysis of the association between sivelestat sodium use and OFFD, adjusting for MV, CRRT, and vasopressor use instead of baseline organ failure.**

| **Variables** | **IRR (95%CI)** | ***p* value** |
| --- | --- | --- |
| Group-Sivelestat administration | 1.20 (1.07-1.35) | **0.002** |
| Treatment era | 0.94 (0.86-1.04) | 0.246 |
| Gender-Female | 0.88 (0.80-0.97) | **0.010** |
| Age | 1.00 (0.99-1.00) | 0.119 |
| Body Mass Index | 1.00 (0.99-1.00) | 0.451 |
| CTSI | 0.99 (0.97-1.00) | 0.266 |
| Organ support |  |  |
| Vasopressor use | 1.01 (0.67-1.53) | 0.962 |
| MV | 0.90 (0.69-1.18) | 0.445 |
| CRRT | 1.11 (0.77-1.61) | 0.573 |
| SOFA Score | 0.93 (0.90-0.95) | **<0.001** |
| APACHE II Score | 0.99 (0.98-1.00) | 0.338 |
| CRP | 0.99 (0.99-1.00) | 0.023 |
| CTSI, computed tomography severity index; MV, mechanical ventilation; CRRT, continuous renal replacement therapy; SOFA, Sequential Organ Failure Assessment; APACHE, acute physiology and chronic health evaluation; CRP, C-reactive protein, IRR, incidence rate ratio. | | |

**Supplementary Table 4.** Proportion of missing data per inflammatory marker.

| Variable | Missing frequent | Missing percentage, % |
| --- | --- | --- |
| Baseline CRP | 0 | 0 |
| Day 3 CRP | 29 | 8.5 |
| Day 5 CRP | 72 | 21.1 |
| Day 7 CRP | 122 | 35.8 |
| Baseline IL-6 | 0 | 0 |
| Day 3 IL-6 | 198 | 58.2 |
| Day 5 IL-6 | 219 | 64.4 |
| Day 7 IL-6 | 244 | 71.7 |
| Baseline PCT | 0 | 0 |
| Day 3 PCT | 146 | 42.9 |
| Day 5 PCT | 183 | 53.8 |
| Day 7 PCT | 214 | 62.9 |
| CRP, C-reactive protein; PCT, procalcitonin; IL, interleukin. | | |

**Supplementary Table 5. Adverse events.**

| **Adverse event** | **Sivelestat group (n=60)** | **Non-Sivelestat group (n=280)** |
| --- | --- | --- |
| Any adverse event, n (%) | 19 (31.7) | 98 (35.0) |
| Hepatic dysfunction, n (%) | 13 (21.7) | 78 (27.9) |
| Renal dysfunction, n (%) | 10 (16.7) | 41 (14.6) |
| Thrombocytopenia, n (%) | 8 (13.3) | 24 (8.6) |
| Platelet count increase, n (%) | 2 (3.3) | 21 (7.5) |
| Allergic reaction, n (%) | 0 (0.0) | 0 (0.0) |
| Secondary infection, n (%) | 0 (0.0) | 0 (0.0) |
| Treatment discontinuation, n (%) | 0 (0.0) | 0 (0.0) |

**Supplementary Figure.** Summaries of the balance of variables before and after propensity score matching.

PSM, propensity score matching; RAC, revised Atlanta classification; SAP, severe acute pancreatitis; CTSI, computed tomography severity index; SOFA, Sequential Organ Failure Assessment; APACHE, Acute Physiology and Chronic Health Evaluation; WBC, white blood cell; CRP, C-reactive protein; PCT, procalcitonin; IL, interleukin; MV, mechanical ventilation; CRRT, continuous renal replacement therapy.
